# Lithium disilicate posterior overlays: clinical and biomechanical features

**DOI:** 10.1007/s00784-019-02972-3

**Published:** 2019-06-14

**Authors:** Malchiodi Luciano, Zotti Francesca, Savoia Michela, Moro Tommaso, Albanese Massimo

**Affiliations:** 1 Department of Surgical Sciences, Paediatrics and Gynecology, University of Verona, Policlinico “Giovanni Battista Rossi” Piazzale Ludovico Antonio Scuro, 10, 37134 Verona, Italy; 2 Verona, Italy; 3 Vicenza, Italy

**Keywords:** Lithium disilicate, Posterior single tooth restorations, Thickness, Dental tissue

## Abstract

**Objectives:**

The aim of this study was to evaluate the survival rate of lithium disilicate overlays in increasing occlusal vertical dimension (OVD) in the setting of minimally invasive techniques and the restoration thicknesses at different tooth sites.

**Materials and methods:**

This is an observational study evaluating 43 lithium disilicate overlays (Lithium IPS e.max Press, Ivoclar Vivadent) on 8 patients, prepared with minimally invasive criteria over a follow-up period between 19 to 45 months (mean follow-up of 32 months). Occlusal vertical dimension’s increase was planned using occlusal treatment plan and diagnostic wax-up. Prior to adhesive cementation, restoration thicknesses were measured with a caliber. The survival rate was calculated by Kaplan-Meier analysis.

**Results:**

Restoration survival rates at 32 months were 97.7%. One infiltration was observed, no cases of fracture occurred. The greatest thickness in monolithic restorations was detected in the cusp sides of teeth, whereas the thinnest was highlighted in the central fossa. The average amount of dental tissue removed during preparation was 0.98 mm in non-functional cusps, 0.88 mm in functional cusps, and 0.57 mm in the central fossa.

**Conclusions:**

Lithium disilicate posterior overlays show an excellent complication-free survival rate, and the material allows for conservative restorations with minimum thickness.

**Clinical relevance:**

Monolithic lithium disilicate overlays feature a satisfying 32-month survival rate. The technique allows to perform restorations with a minimal removal of dental tissue, while limiting fractures over time. Its esthetical performance is excellent.

## Introduction

The evolution of mechanical properties in restorative materials and adhesive cementation has led to the development of minimally invasive preparation criteria, which allow to preserve significant amounts of dental tissue with a consequent maximum reinforcement of dental elements. Importantly, the new materials have also contributed to overcoming the intrinsic problems related to the all-ceramic system, such as susceptibility to fracture and wear of antagonists. In recent years, the use of indirect adhesive restorations has also been extended to posterior teeth [1] achieving excellent results in terms of marginal closure, esthetic results, and reinforcement of the residual tooth structure, especially where cusps are covered [1, 2].

Among these new materials, lithium disilicate is one of the most promising, thanks to its high mechanical strength, extraordinary versatility, and excellent optical properties. Although glass ceramics are commonly indicated for esthetic restorations in the anterior area [3], the excellent biomechanical characteristics of lithium disilicate [4] make the material also suitable for monolithic inlays in the posterior teeth. In particular, by using lithium disilicate, posterior loading requirements can be met with a more conservative restoration, with a thickness of just 1.0 mm, compared with the 1.5 to 2.0 mm commonly recommended for porcelain restoration [5]. Moreover, disilicate overlays allow to modify tooth occlusal surface and carry out wider rehabilitations in complex oral treatment plans. This feature makes corrections of occlusal relationship or increases in vertical dimension possible.

Hence in light of current evidence in the literature and our experience with this material, we aimed to evaluate the survival rate of lithium disilicate overlays performed to increase occlusal vertical dimension (OVD) by means of minimally invasive intervention and then evaluate the thickness of restorations in different teeth sites.

## Materials and methods

The present study was an observational descriptive study evaluating 43 monolithic lithium disilicate overlay restorations (8 patients), to increase occlusal vertical dimension. The occlusal plane modifications had been planned during the treatment-planning phase. Restorations were then performed using different techniques such as overlays, crowns, bridges, and veneers, in order to provide a comfortable occlusion for patients and provide complete oral rehabilitation; however, only the overlays were included in this study. An increase in vertical occlusion dimension had been required in all patients to fix temporomandibular joints and muscle relationships after dental wear or altered occlusal conditions. Inclusion criteria were overlay of posterior restoration, restoration of altered occlusal conditions and wear of teeth, teeth receiving endodontic treatment, and composite preceding restorations. Exclusion criteria were poor oral hygiene, active periodontitis, probing depths more than 4 mm, and implant-supported restorations crowns [6].

Evaluation was limited to lithium disilicate overlays; the variables assessed were restoration thickness, average amount of dental tissue removed during preparation, and size of occlusal increase. Values collected were compared with those in the current literature. An observation period ranged between 19 and 45 months was considered (between December 2014 and January 2018) with an average time of follow-up of 32 months. Survival was defined as restoration being in situ with or without complications for the entire observation period. Assessment of survival was carried out for each restoration by the same trained examiner.

The 43 overlays were performed by using IPS e.max Press (Ivoclar Vivadent Manufacturing SRL, BZ, Italy) applied to 15 maxillary and 28 mandibular, following a precise cementation protocol.

At the first visit, an alginate dental impression was taken in each patient and cast models and diagnostic wax-up were made to plan the amount of occlusal rise needed for each dental element. By mounting on adjustable dental articulator dental casts, worn surfaces of teeth needing treatment were waxed and the amount of occlusal increase was planned aforetime on cast models.

**Fig. 1:**
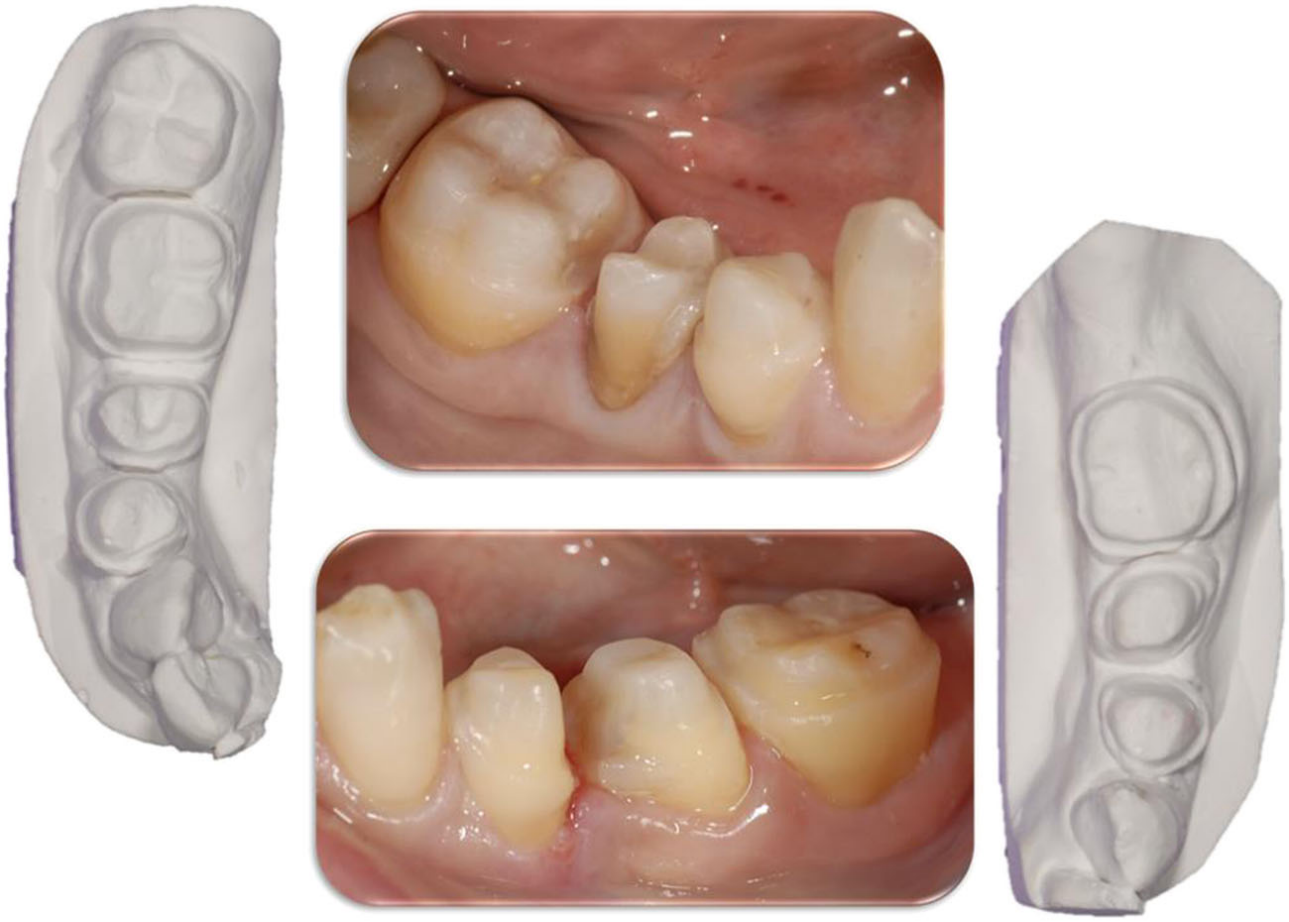
Minimal invasive preparations of teeth (clinical and cast models views)

Cavities were prepared complying with dental anatomy and maintaining the margins above the gum line in agreement with minimally invasive criteria in order to preserve tissues, avoid traumas, and reach a better prognosis for dental elements prepared (Fig. 1, Table 1) [7, 8].

**Table 1:** Summary of criteria and conclusions about minimal invasive preparations

Study	Analyzed thicknesses	Conclusions
Ma Li, Petra C. Guess, Yu Zhang (2013) *Load-bearing properties of minimal-invasive monolithic lithium disilicate and zirconia occlusal onlays: finite element and theoretical analyses* Dental Materials	0.5 mm 1 mm 2 mm	The load-bearing capacity of lithium disilicate bonded to enamel can approach 75% of that of zirconia with thicknesses between 0.7 and 1.4 mm. The fracture load of ultra-thin inlays supported by enamel was comparable with standard supported by dentin.
Petra C. Guess (2013) *Influence of preparation design and ceramic thicknesses on fracture resistance and failure modes of premolar partial coverage restorations* The Journal of Prosthetic Dentistry	0.5 mm 1 mm 2 mm	All premolar pressed lithium disilicate glass ceramic partial coverage restorations revealed failure loads exceeding physiologic mastication forces. The reduction of thicknesses did not impair fracture resistance of onlay restorations.
Fradeani et al. (2012) *Esthetic rehabilitation of a severely worn dentition with minimally invasive prosthetic procedures (MIPP)* The Journal of Prosthetic Dentistry	0.8–1 mm	The dental tissue preservation and the restoration bonding on enamel guarantee sufficient resistance to restoration, even in the presence of minimum thicknesses.
Morakot Piemjai et al. (2007) *Compressive Fracture Resistance of Porcelain Laminates Bonded to Enamel or Dentin with Four Adhesive Systems* Journal of Prosthodontics	0.5 mm 1 mm 2 mm	The minimum enamel preparation for the 0.5-mm porcelain thickness achieved better fracture resistance for enamel-bound porcelain compared with a deeper dentin preparation to 1.0-mm thickness.

Overlays were adhesively cemented using Variolink Esthetic (Ivolclar Vivadent Manufacturing SRL, BZ, Italy). The inner layer of restorations was etched by hydrofluoric acid 9.6% (ENA etch, Micerium SPA, Avegno, Italy) for 30 s and then rinsed and dried. A silane coupling agent (Monobond, Ivoclar Vivadent Manufacturing SRL, BZ, Italy) was applied for a minute onto the inner surfaces and then air-dried. Subsequently, a layer of adhesive (Adhese Universal, Ivoclar Vivadent Manufacturing SRL, BZ, Italy) was placed avoiding polymerization in order to not increase the thickness in the tooth/restoration surface.

The operative field was isolated by use of a rubber dam; the dental cavity was then conditioned with 37% orthophosphoric acid (ENA total etch phosphoric acid 37%, Micerium SPA, Avegno, Italy), 30 s for enamel, and 15 s for dentin, being careful to protect contiguous teeth. Dental surfaces were then abundantly rinsed and air-dried. Etch & rinse bonding was placed (Adhese Universal VivaPen, Ivoclar Vivadent Manufacturing SRL, BZ, Italy) but not polymerized to avoid incongruous thicknesses on the tooth/restoration surface. Cementation was performed with Variolink Esthetic (Ivoclar Vivadent Manufacturing SRL, BZ, Italy) placed on the inner surface of the overlay. The overburden was removed with a brush before polymerization (1 min per surface), and margins were finished with a rubber pad. Occlusion was assessed and polishing was carried out.

### Evaluation

Each complication was considered a statistical event; cumulate survival was recorded using Kaplan-Meier analysis.

The thickness of restoration is represented by the sum of extent of the occlusal increase and the amount of dental tissue removed during the preparation. Appraisal of restoration thickness included measurement with a thickness gauge and measurements of the functional cusp, non-functional cusp, and the central point of the fossa of prosthetic restorations. For each of these landmarks, the mean and standard deviation were calculated.

Measurement of molar overlay thicknesses of functional and non-functional cusps was performed for both the mesial portion and the distal portion; these were then expressed as a single value. The results obtained from the thickness analysis were compared with those present in the literature. A further evaluation on overlays thickness was carried out by subgroup characterization: very thin, thin, medium, and thick.

The amount of dental tissue removed was measured using a cutter of known size during preparation, and the thickness of overlays was measured using a millimetric thickness gauge. The extent of the occlusal rise was obtained by the difference between these two values. Subsequently, the ratios of extent of occlusal increase and of amount of dental tissue removed were determined within the overlay thickness, and a *t* test was performed to compare the meanings of these two elements.

All values are expressed as mean ± standard deviation. Statistical tests were considered significant for *P* ≤ 0.05.

All statistical analyses were performed using Statistical Package for Social Sciences Version 22.0 (SPSS Inc., Chicago, IL).

## Results

**Fig. 2:**
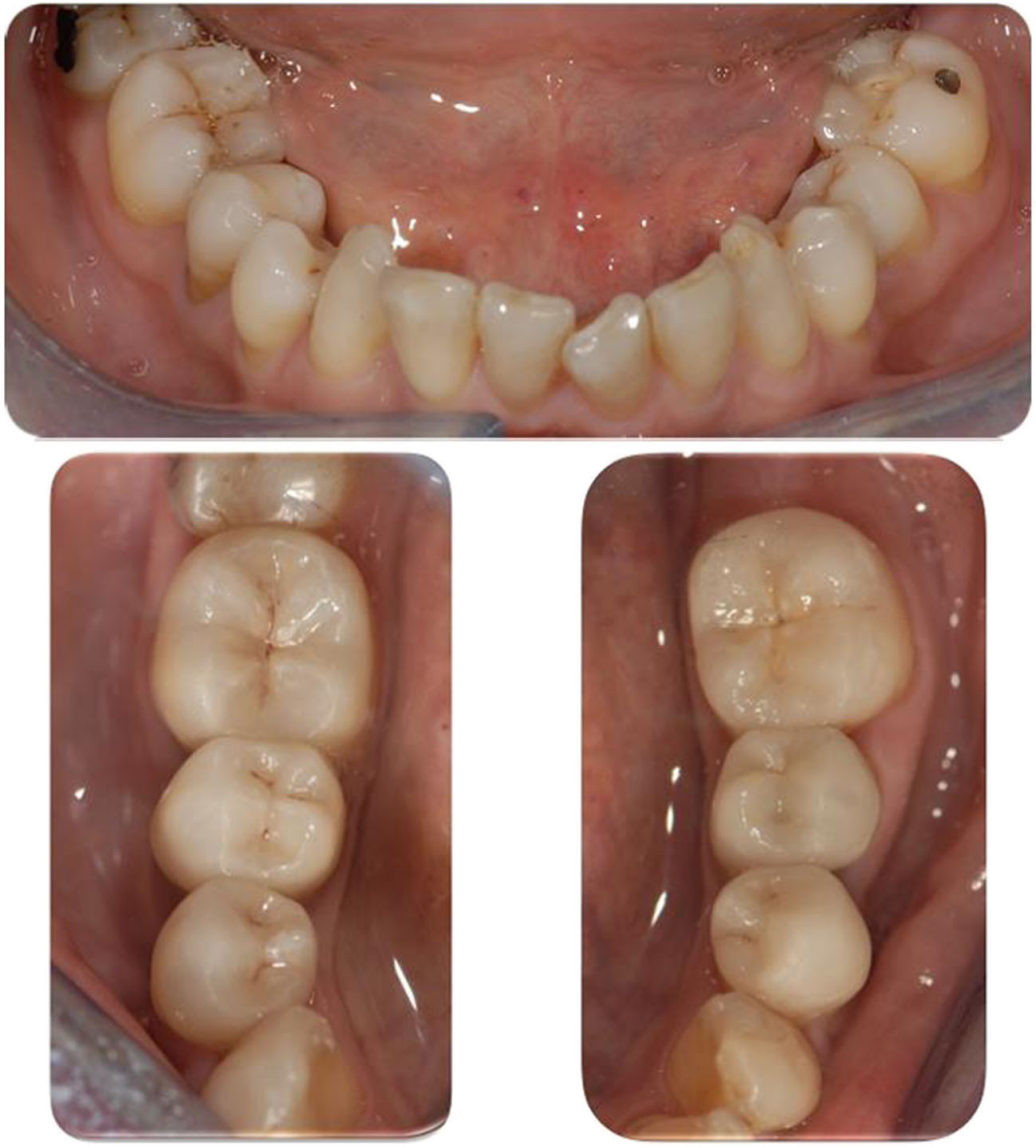
Lower arch, before and after repair

In this study, 43 overlays were performed in 8 patients. The study enrolled patients aged 47 to 67 years (average 57.9 ± 7.22 y.o.). The follow-up was 32 months (range 19–45 months). Eighteen molars (42%) and 25 premolars (58%) had been restored, in detail, 15 (35%) overlays in the maxillary arch and 28 (65%) in the mandibular (Fig. 2).

**Graph 1:**
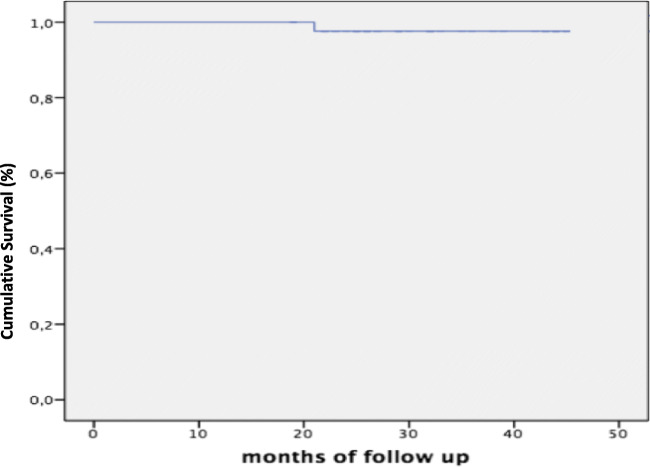
Kaplan-Meier diagram of survival rate on average follow-up

A case of infiltration occurred, but no cases of dental fracture or overlay fracture were noticed. The success rate was 97.7% as calculated on average follow-up (Graph 1).

The single infiltration observed received endodontic treatment; the margin was reshaped and a composite restoration was carried out.

Values obtained by thickness analysis are shown in Table 2.

**Table 2:** Mean values of thickness (mm) ± standard deviations at different landmarks, statistical significance, and clinical results

	Thickness in non-functional cusps	Thickness in functional cusps	Thickness in the central point of the fossa
Mean ± SD	2.06 ± 0.49	1.94 ± 0.39	1.19 ± 0.31
Reference value	≥ 0.7 mm	≥ 0.7 mm	≥ 0.7 mm
Null hypothesis H0	T-NFC < 0.7 mm	T-FC < 0.7 mm	T-CF < 0.7 mm
Significance of t-test	< 0.001	< 0.001	< 0.001
Result	≥ 0.7 mm	≥ 0.7 mm	≥ 0.7 mm

*T* thickness, *NFC* non-functional cusps, *FC* functional cusps, *CF* central point of the fossa

The value, expressed as mean ± standard deviation, is compared with the reference values reported in the literature (*t* test).

**Graph 2:**
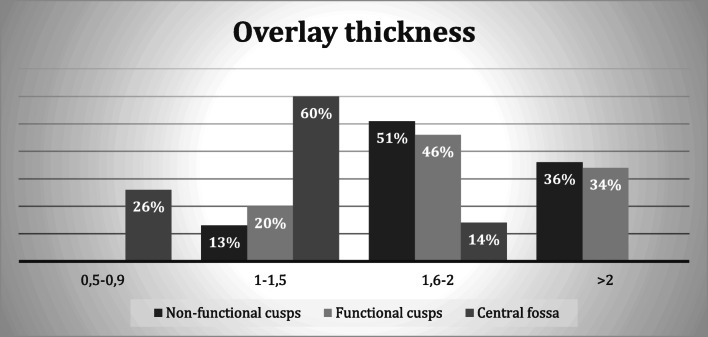
Representation of thickness (mm) at different landmarks of restoration

Graph 2 shows the percentage of different thickness of restorations, very thin (0.5–0.9 mm), thin (1–1.5 mm), medium (1.6–2 mm), thick (> 2 mm), at the restorations landmarks.

On functional and non-functional cusps, the thickness was remarkably higher than 1.5 mm (87% and 80%, respectively). Instead, in the central point of the fossa, the thickness was found to be thinner (86% is less than 1.5 mm).

**Graph 3:**
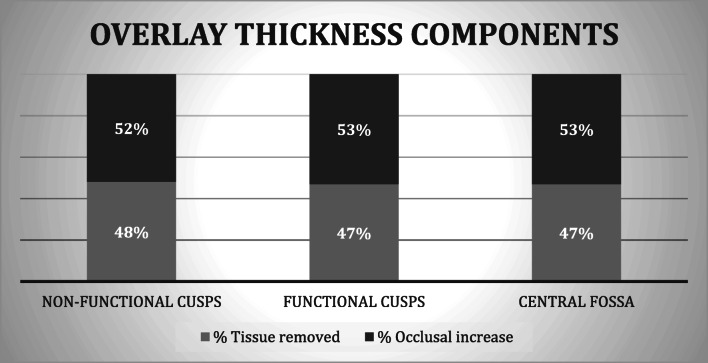
Average ratio (percentage) of tissue removed and occlusal increase within the thickness of overlays

The ratios of the amount of dental tissue removed and occlusal increase were defined in relation to the thickness at each landmark, and percentage of each value was calculated (Graph 3).

Table 3 describes the extent of these components in detail.

**Table 3:** Ratio of occlusal increase and removed tissue, *P* values

Percentage of occlusal increase	NFC	52%
	FC	53%
	CF	53%
Percentage of tissue removed	NFC	48%
	FC	47%
	CF	47%
*P* value in NFC = 0.366 *P* value in FC = 0.112 *P* value in CF = 0.128		

*FC* functional cusps, *NFC* non-functional cusps, *CF* central point of the fossa

Results of the *t* test performed to assess ratios of removed tissue and occlusal increase did not evidence any statistically significant difference in percentage values of tissue removed and of occlusal increase in any landmark (*t* test ≤ 0.05), which indicates that, within the thickness of overlay, the amount of tissue removed is likely to be the same as occlusal increase entity (Table 3).

**Graph 4:**
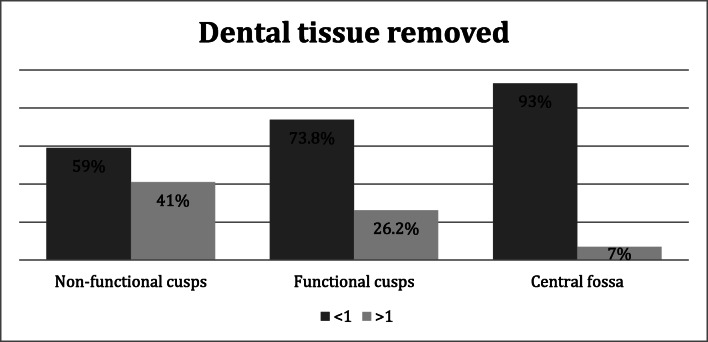
Amount (percentage) of dental tissue removed during preparation (mm)

Graph 4 shows that the depths of most of preparation are less than 1 mm, according to minimum invasive criteria. The amount of tissue removed was less than or equal to 1 mm in 59% of preparations of the non-functional cusps, 74% of the functional cusps, and 93% of the central fossa.

## Discussion

The aim of the present study was to evaluate specific characteristics of restorations, specifically in the setting of increasing vertical dimension in patients with worn dentition. Particular attention was set on the possibility of performing minimally invasive preparations and evaluating the thicknesses of the material used in terms of restoration survival.

Our choice towards lithium disilicate was driven by its slightly higher hardness and resistance compared with those of tooth enamel, which makes it suitable for an increase in vertical dimension. This allows to maintain the material’s wear and tear similar to that occurring with the original enamel, thus limiting, at least in part, the gnathologic consequences in many cases typical of extended occlusions with protheses.

Overall results are encouraging. As expected, restoration survival rates in our study were in agreement with current data from the literature on posterior restorations [7, 9], with 97.7% at 32 months and no restoration fractures observed [7, 9, 10]. Moreover we were able to perform a minimally invasive preparation, while maintaining adequate thicknesses and avoiding pointless loss of dental tissue, as suggested by current evidence.

Studies in the literature have estimated the depth of preparation for traditional monolithic ceramic systems needed to provide resistance to tensile stress of inner cementation surfaces under occlusal load to be approximately 1.5–2 mm [1, 3, 11]. With specific reference to for IPS e.max Press lithium disilicate ceramic, most manufacturers recommend a minimum thickness of 1.5 mm; however, such indications are mostly based on in vitro tests and do not ensure enforceable clinical results [12]. In recent years several studies have evaluated the resistance of minimum thicknesses of monolithic lithium disilicate IPS e.max Press restorations and have found that the reduction in thickness does not affect the material bending strength and warrants its use in preparations with minimal dental tissue removal (Table 1). The minimum thicknesses assessed in our study were below the 1.5 mm recommended by the traditional literature for ceramic restorations. In our study, we had removed 1.1 mm in the cusps and 0.7 mm at the fossa, yet no restoration fractures were observed. As to overlay thicknesses, 26% at the fossa were between 0.5 and 0.9 mm and 60% between 1 and 1.5 mm. In the cusps, the thicknesses were higher: only 13% and 20% for non-functional and functional cusps, respectively, were less than 1.5 mm. Preservation of residual tissues in long-term element survival—especially where caries or erosion have weakened the dental structure—is fundamental [8, 13] and entails fewer traumas and better prognosis [8]. A crucial and critical point about this concerns the occurrence of higher loading failure rate, as the supporting tooth structure is predominately made up by enamel, which has a higher elastic module compared with those of dentin [14, 15]. Disilicate adhesive cementation plays a key role in this context: enamel and lithium disilicate have a very similar elastic module (80 GPa—which rises to 91 GPa on the occlusal surface—and 95 GPa), and this allows to develop less tension stresses during chewing load, reducing the threat of ceramic fracture [16]. The fracture risk in thin restorations cemented on enamel is therefore comparable with thicker restorations cemented on dentin; moreover, enamel-cemented restorations show less mechanical complications over the years [17].

In an ideal tooth preparation, the aspect that needs most careful evaluation is the amount of dental structure removal required by the material being used. In addition to this, other important clinical criteria that need to be taken into account are residual tooth condition, esthetic and functional aspects, tooth orientation, and occlusion rehabilitation planning.

In most of the preparations analyzed in our study, the amount of dental tissue removed is less than or equal to 1 mm, according to minimum invasive criteria.

However, overlay thicknesses analyzed in our study did not match only the amount of dental tissue removed, but they also depended on required occlusal increase, which we had calculated during the treatment planning phase in order to reestablish a correct occlusal relationship, or an occlusal vertical dimension (OVD) decreased due to dental abrasion [18]. To gain further insight on the matter, in fact we further analyzed our restorations and determined different thickness ratios. On the non-functional cusps, the percentage of tooth removal needed was 48% for ceramic thickness and 52% for occlusal increase; on the functional cusps and the central fossa, the values of dental tissue removed and occlusal increase were, respectively, 47% and 53% in both cases. This explains how the need for obtaining an occlusal increase allows the clinician to carry out more conservative preparations, maintaining the structure of the dental elements and performing adhesion in the enamel.

## Conclusions

Lithium disilicate IPS e.max Press confirmed to be a reliable material for monolithic restorations yielding a highly satisfying survival at 32 months. Its biomechanical characteristics allowed us to work on minimal thicknesses values of 0.7 mm without affecting the strength.

Future studies extending the follow-up period could be useful to assess the mechanical behavior in these conservative restorations over time and evidence any changes in esthetic performances.
